# Meniscus-Related Videos on TikTok Are Widely Viewed and Shared but the Educational Quality for Patients Is Poor

**DOI:** 10.1016/j.asmr.2024.100927

**Published:** 2024-03-19

**Authors:** Riccardo D’Ambrosi, Timothy E. Hewett

**Affiliations:** a IRCCS Ospedale Galeazzi – Sant’Ambrogio, Milan, Italy; b Università degli Studi di Milano, Dipartimento di Scienze Biomediche per la Salute, Milan, Italy; c Marshall University, Department of Orthopaedics, Huntington, West Virgina, U.S.A.

## Abstract

**Purpose:**

To evaluate the quality of meniscus-related TikTok videos to better understand their value for patient education.

**Methods:**

The term “meniscus” was used as the key word for an extensive online search of video content on the TikTok on November 14, 2023. The first 100 videos were used for analysis. The duration of the videos and the number of likes, shares, and views were recorded for each video. Furthermore, videos were categorized based on the source (health workers, private user), the type of subject (patient experience, physical therapy, anatomy, clinical examination, surgical technique and injury mechanism), type of content (patient experience/testimony, education, rehabilitation), and the presence of music or voice. The quality and reliability assessments of video contents were conducted using the DISCERN instrument, the *Journal of the American Medical Association* benchmark criteria, and Global Quality Score.

**Results:**

Of the 100 videos included in this study, 62 (62%) videos were published by health workers and 38 by private users (38%). Most of the information regarded patient experience (36, 36%), followed by physical therapy (32, 32%), anatomy (14, 14%), clinical examination (8, 8%), surgical technique (6, 6%), and injury mechanism (4, 4%). Video content reported patient experience in 39 (39%) videos, rehabilitation in 31 (31%) videos, and education in the remaining 30 (30%). The mean length of the videos was 39.12 ± 49.56 seconds. The mean number of views was 1,383,001.65 ± 5,291,822.28, whereas the mean numbers of comments, likes and shares were 408.53 ± 1976.90, 54,763.43 ± 211,823.44 and 873.70 ± 2,802.01, respectively. The mean DISCERN score, *Journal of the American Medical Association* benchmark criteria score, and Global Quality Score were 17.93 ± 5.07, 0.24 ± 0.47, and 1.15 ± 0.41, respectively.

**Conclusions:**

Meniscus-related videos on TikTok are widely viewed and shared but the overall educational value to patients is poor.

**Clinical Relevance:**

As patients increasingly use social media to learn about their conditions, it is important for orthopaedic health care professionals to understand the limitations of TikTok videos addressing the meniscus as potential sources of information for their patients.

Meniscal lesions are a prevalent type of knee injury that occur within the joint.^1^ As a result, they are a leading cause of surgical interventions in orthopaedic surgery. The average yearly occurrence of meniscal injuries in the United States has been documented to be 66 per 100,000 individuals, with 61 of these cases leading to either partial or complete removal of the meniscus.^2^ The modifications in the mechanics of sporting activities involving pivoting movements over the past few decades have led to a rise in the incidence of meniscus injuries.^3^, ^4^, ^5^, ^6^, ^7^ Acute meniscal injuries are prevalent (40%-80%), particularly when combined with anterior cruciate ligament (ACL) injuries.^3^^,^^8^^,^^9^

The preservation of the meniscus is crucial for maintenance of the overall health of the knee joint. Unaddressed meniscus tears result in sporadic pain, swelling of the joint, recurring mechanical issues (such as clicking, catching, and giving way), and consequently, a substantial decline in the quality of life, particularly in predominantly young and physically active individuals.^10^^,^^11^ Over time, meniscus tears can lead to the development of joint deterioration and ultimately knee osteoarthritis (OA), which is accompanied by discomfort, limited mobility, and the requirement for knee arthroplasty.^11^^,^^12^

TikTok is a social media network that facilitates the sharing, creation, and consumption of user-generated content. The paradigm of vertical scrolling, shortform, video-based content is straightforward to understand, conveniently accessible, and searchable through the use of hashtags to emphasize important topics.^13^ It is important that patients receive accurate information about these debilitating injuries. Since its introduction in the United States in August 2018, the site has undergone rapid and growth, currently reporting 150 million monthly users who are based in the United States as of February 2023.^13^ Considering the well-documented effectiveness of video-based patient education in enhancement of patient satisfaction, clinicians should consider the use of TikTok as a means for patients to comprehend medical information. The potential for a video to become viral and its attractiveness can greatly affect a patient’s impression of the information offered on platforms such as TikTok.^14^ Regrettably, the extensive propagation of erroneous health information on social media has become a substantial public health issue. Health misinformation refers to health-related assertions that are based on anecdotal evidence, are blatantly untrue, or are misleading because of a lack of robust scientific support.^15^

The quality of information and the characteristics of TikTok videos, as well as their potential conflicts of interest, remain mostly undisclosed. This underscores the necessity of promoting reliable posts containing evidence-based health information to reach a wide audience, especially considering the challenge of controlling the spread of disinformation.^16^

An increasing number of patients and family members are turning to social media platforms like TikTok to find medical information, with as many as 96% of parents using social media for health education.^17^ TikTok defines misinformation as erroneous or false content and prohibits the sharing of medical misinformation that could impair someone’s physical health, as stated in their online community guidelines.^18^ This highlights the necessity of infodemiology, the field of managing health information, and the importance of good communication regarding medical issues alongside the growth of social media.^15^

The purpose of this study was to evaluate the quality of meniscus-related TikTok videos to better understand their value for patient education. The hypothesis was that the video content on this platform would not provide adequate and valid information for patient education.

## Methods

The current study was exempt from institutional review board approval and did not require approval by the participants, as no data of human beings were used. The current study focused on meniscus videos on the TikTok social media platform. The term “meniscus” was used as the key word for an online search of video content on the TikTok video platform (November 14, 2023).

The app uses an algorithm-based, highly personalized feed designed to deliver videos tailored to individual users, considering factors like your interests, who you follow, and what’s trending. As a result, there is simply no way to sort videos according to the date they were posted, topic, or number of likes or views.

Out-of-topic, non-English, and duplicated videos were excluded from the analysis. The first 100 videos were used for analysis. The duration of the videos and the number of likes, shares, and views were recorded for each video. Furthermore, videos were categorized based on the source (health workers or private user), type of information (patient experience, physical therapy, anatomy, clinical examination, surgical technique, injury mechanism), video content (patient experience/testimony, rehabilitation, education), and the presence of music or voice.

The quality and reliability assessments of video contents were conducted using the DISCERN instrument, the *Journal of the American Medical Association* (JAMA) benchmark criteria, and the Global Quality Score (GQS) by 2 experienced knee surgeons.^19^, ^20^, ^21^, ^22^, ^23^, ^24^, ^25^, ^26^

### Assessment Tools of Video Reliability, Validity, and Quality

##### DISCERN Instrument

The DISCERN tool is an assessment scale developed for patients and providers to assess the reliability and quality of information. The tool, which consists of 16 items in total, is divided into 3 parts. Items 1 through 8 form the first part and measure the reliability of the information. Items 9 through 15 form the second part, measuring the quality of the information, and the last section consists of a single item with an overall quality rating (Item 16). DISCERN uses a 5-point Likert scale. For evaluation of the first 15 items, 1 point indicates “no,” and 5 points indicates “yes”; the responses are evaluated within this range. For the 16th item, 1 point indicates “low quality with serious or extensive deficiencies,” 5 points indicates “high quality with minimum-wax deficiencies,” and the responses are evaluated within this range. The total DISCERN score was calculated as the sum of the first 15 items, with a minimum score of 15 and a maximum score of 75. The reliability and quality of the information are characterized by an increase in scores, where a score of 15 to 27 points indicate “very poor,” 28 to 38 points indicate “poor,” 39 to 50 points indicate “medium,” 51 to 62 points indicate “good,” and 63 to 75 points indicate “excellent.” DISCERN is freely accessible at http://www.discern.org.uk.^19^^,^^20^^,^^24^

##### JAMA Benchmark Criteria

The JAMA benchmark criteria instrument is one of the leading tools used to evaluate medical information obtained from online sources. It includes 4 criteria, authorship, attribution, disclosure, and currency, with a value of 1 point each and a total score of 4 points. In the JAMA evaluation, a score of 0 to 1 point represents insufficient information, a score of 2 to 3 points represents partially sufficient information, and a score of 4 points represents completely sufficient information.^25^^,^^26^

##### Global Quality Score

GQS is a scoring system that can be used to assess a video in terms of its instructive aspects for viewers. It allows for the evaluation of quality, streaming, and ease of use of information presented in online videos. In the evaluation of GQS, a score of 1 indicates that the video has the poorest quality and is not useful for viewers, whereas a score of 5 indicates that the video has excellent quality and is very useful for viewers.^21^^,^^22^

### Statistical Analysis

Descriptive statistics were presented for all video characteristics included video sources, video content, type of video information, as well as for the outcomes (i.e., DISCERN, JAMA, and GQS). Categorical variables were shown as absolute frequencies and percentages, whereas continuous variables were presented both as mean and standard deviation or median, interquartile range, and range. Correlations between quantitative variables were estimated and tested using the Spearman rank correlation test. A *t*-test or a Wilcoxon-Mann-Whitney *U* test was performed to assess whether outcomes differed by video sources, video content, audio, and type of video information. Bonferroni adjustment was used for multiple comparisons. Categories with greater frequency were selected as reference. For tests and regression models some categories of type of video information, specifically anatomy, clinical examination, surgical technique, and injury mechanism, were grouped in one category, named “other,” because of their low frequency. All tests were 2-tailed, and a *P*-value < .05 was considered to indicate statistical significance. All statistical tests were performed with R (R Foundation for Statistical Computing, Vienna, Austria; https://www.R-project.org).

## Results

Of the 100 videos included in this study, 62 (62%) videos were published by health workers and 38 by private users (38%). Most of the information regarded patient experience (36, 36%), followed by physical therapy (32, 32%), anatomy (14, 14%), clinical examination (8, 8%), surgical technique (6, 6.0%), and injury mechanism (4, 4%). Video content reported patient experience in 39 (39%) videos, rehabilitation in 31 (31%) videos, and education in the remaining 30 (30%). Forty-three (43%) videos used music as the audio background, and 57 (57%) had voice comments.

**Fig 1:**
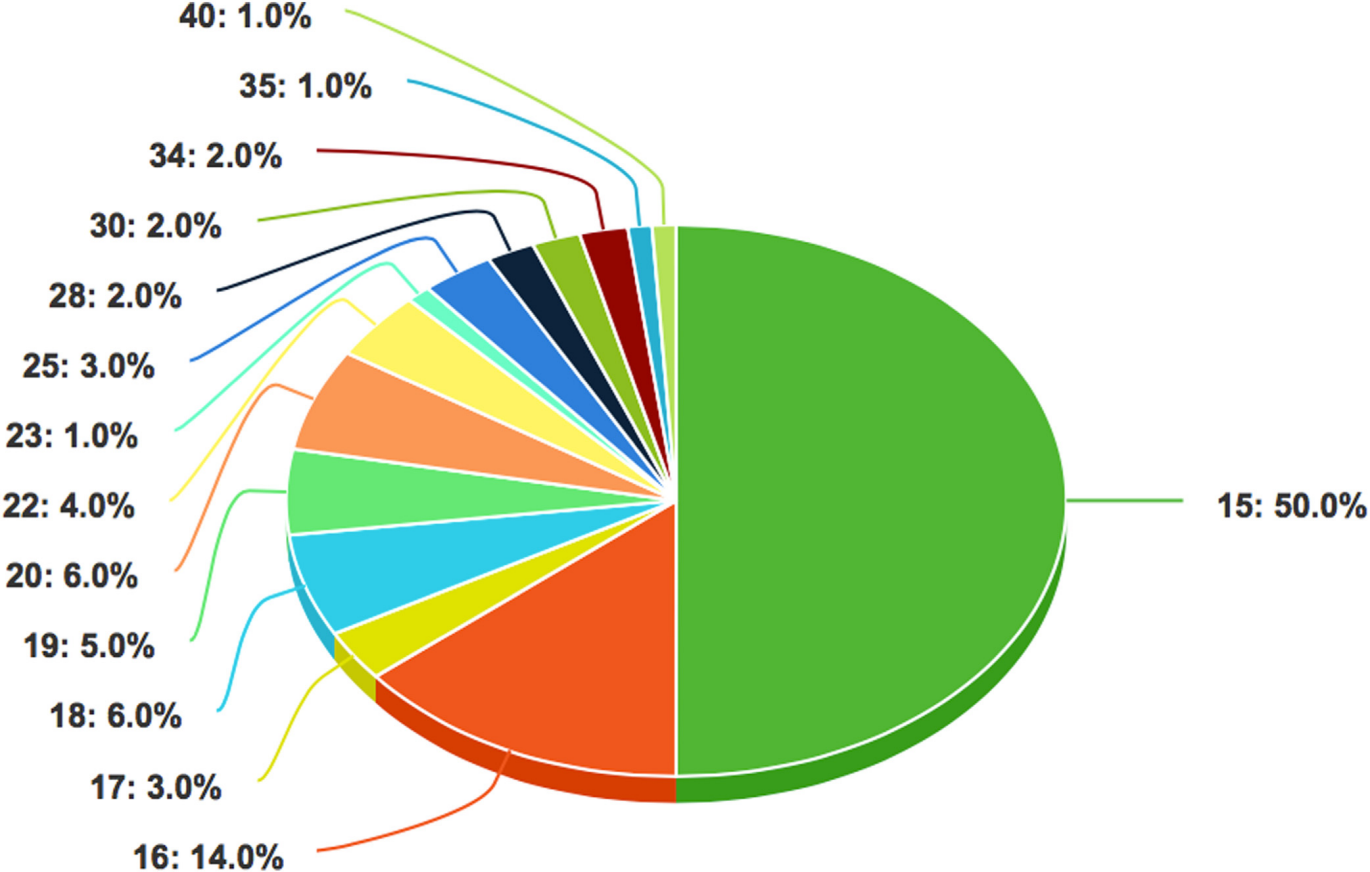
The pie chart shows the distribution of scores in accordance with the DISCERN Instrument. One-half of the videos (50%, 50 videos) show a score of 15, considered to be the worst possible. More than 80% of the videos show a score ≤20, whereas only 1 video (1%, 1 video) demonstrates a score of 40.

**Fig 2:**
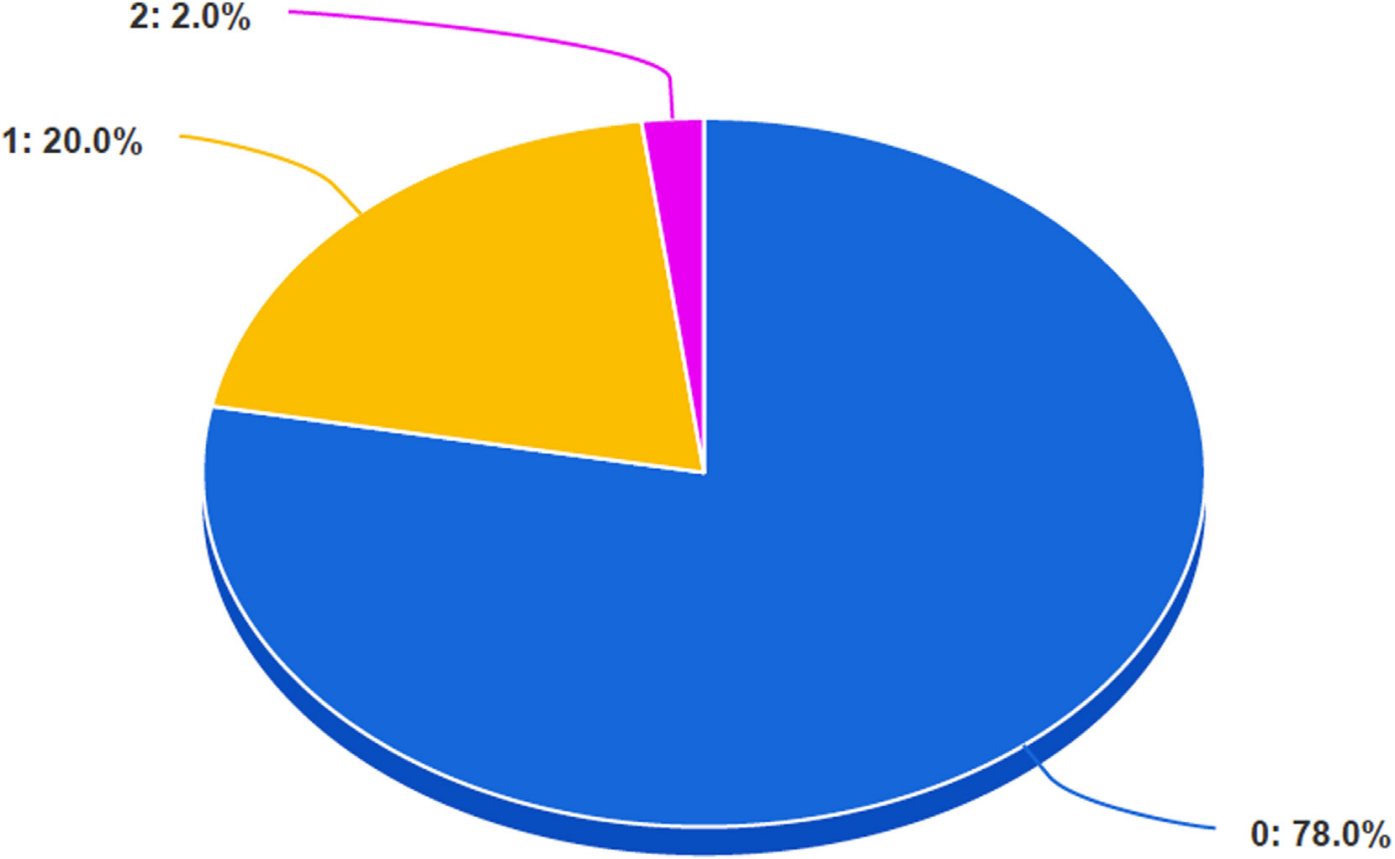
The pie chart shows the distribution of scores in accordance with the JAMA score. More than 75% of the videos show the worst possible result (score equal to 0), whereas only 2% (2 videos) show a sufficient result (score equal to 2). (JAMA, *Journal of the American Medical Association* benchmark criteria)

**Fig 3:**
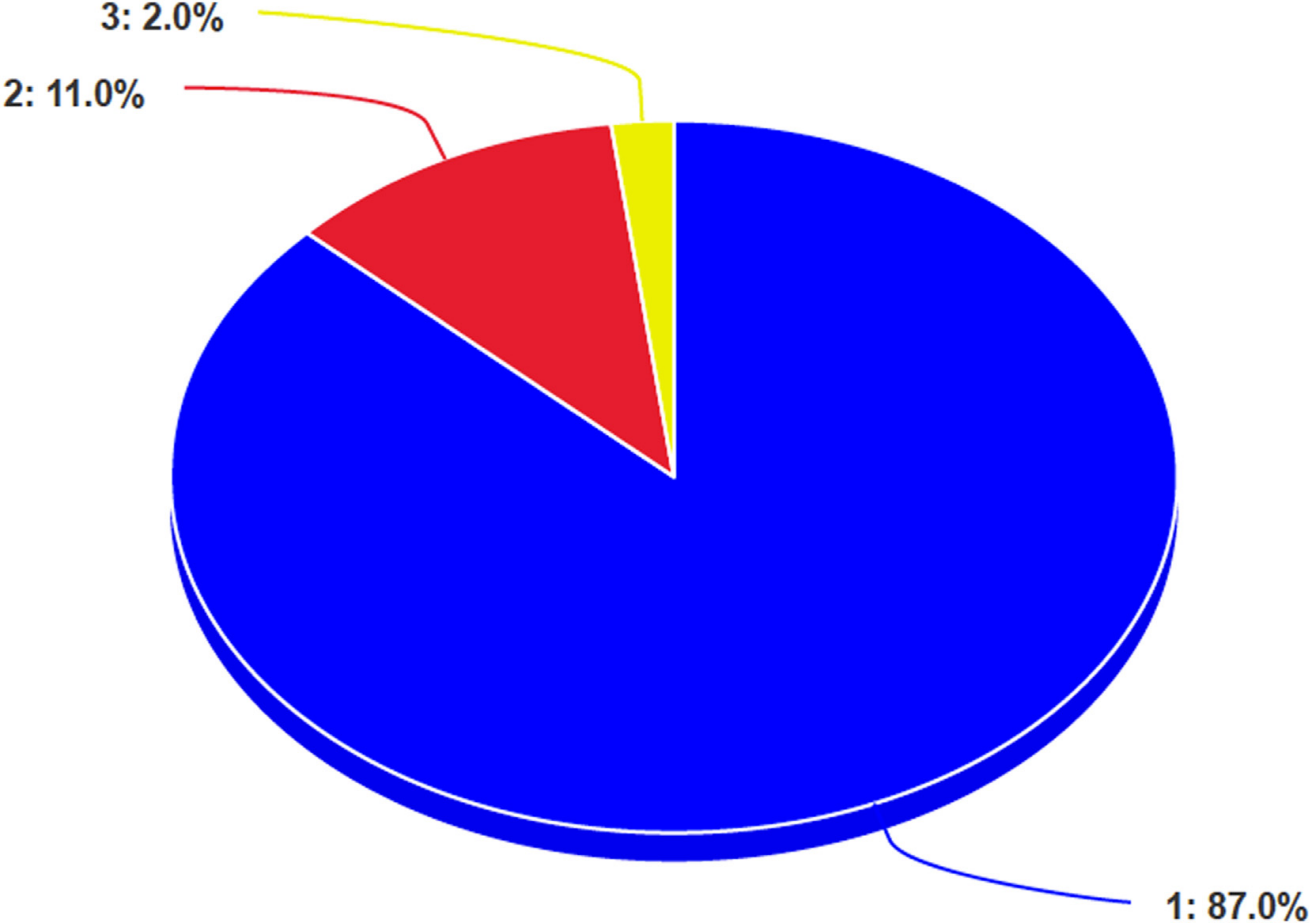
The pie chart shows the distribution of scores in accordance with the GQS scores. Almost all the videos (98%, 98 videos) show a poor result with a score between 1 and 2. Only 2 videos show a fair result (score equal to 3). (GQS, Global Quality Score.)

The mean length of the videos was 39.12 ± 49.56 seconds. The mean number of views was 1,383,001.65 ± 5,291,822.28, whereas the mean numbers of comments, likes, and shares were 408.53 ± 1,976.90, 54,763.43 ± 211,823.44, and 873.70 ± 2,802.01, respectively. The mean DISCERN score, JAMA score, and GQS were 17.93 ± 5.07, 0.24 ± 0.47, and 1.15 ± 0.41, respectively. Detailed results are reported in Table 1 and Fig 1, Fig 2, Fig 3.

**Table 1:** Categorical and Continuous Variables

Variables	n	%
Categorical, N = 100		
Video source		
Health workers	62	62
Private user	38	38
Type of information		
Patient experience	36	36
Physical therapy	32	32
Anatomy	14	14
Clinical examination	8	8
Surgical technique	6	6
Injury mechanism	4	4
Video content		
Patient experience/testimony	39	39
Rehabilitation	31	31
Education	30	30
Audio characteristics		
Music	43	43
Voice	57	57

Continuous	Mean ± SD	Median [IQR; range]
Video characteristics		
Total number of views	1,383,001.65 ± 5,291,822.28	126,300 [44,075-633,500; 1543-36,600,000]
Total number of likes	54,763.43 ± 211,823.44	2,707 [1,104.50-21,600; 52-1,900,000]
Total number of comments	408.53 ± 1,976.90	64 [17.50-199.50; 0-19,500]
Total number of shares	873.70 ± 2,802.01	143 [27-380; 0-25,000]
Video length, s	39.12 ± 49.56	28 [17-46; 5-451]
Video score		
DISCERN	17.93 ± 5.07	15.50 [15-19; 15-40]
JAMA	0.24 ± 0.47	0 [0-0; 0-2]
GQS	1.15 ± 0.41	1 [1-1; 1-3]

GQS, Global Quality Score; IQR, interquartile range; JAMA, *Journal of the American Medical Association* benchmark criteria; SD, standard deviation.

Analysis of videos by video source demonstrated greater value for videos posted by health workers compared with private users for all the scores (*P* < .05). Detailed results are reported in Table 2.

**Table 2:** Score Difference by Video Sources

	Health Workers, n = 62	Private User, n = 38	*P* Value
	Mean ± SD	Mean ± SD	
DISCERN	19.66 ± 5.79	15.11 ± 0.51	<.001∗
JAMA	0.37 ± 0.55	0.03 ± 0.16	<.001∗
GQS	1.24 ± 0.50	1.00 ± 0.00	.003∗

GQS, Global Quality Score; IQR, interquartile range; JAMA, *Journal of the American Medical Association* benchmark criteria; SD, standard deviation.

∗ Statistically significant value.

Analysis of video content showed that videos regarding anatomy, clinical examination, surgical technique, and injury mechanism had greater quality than videos regarding patient experience and physical therapy (*P* < .05). Physical therapy videos showed greater DISCERN scores compared with patient experience (*P* < .05). Detailed results are reported in Table 3.

**Table 3:** Score Difference by Type of Information

				Pairwise Comparisons		
	Patient Experience, n = 36	Physical Therapy, n = 32	Other,∗ n = 32	Patient Experience Versus Physical Therapy	Patient Experience Versus Other	Physical Therapy Versus Other
	Mean ± SD	Mean ± SD	Mean ± SD	*P* Value	*P* Value	*P* Value
DISCERN	15.11 ± 0.52	17.41 ± 3.55	21.62 ± 6.75	<.001†	<.001†	.028†
JAMA	0.03 ± 0.17	0.12 ± 0.34	0.59 ± 0.61	.393	<.001†	.002†
GQS	1.00 ± 0.00	1.03 ± 0.18	1.44 ± 0.62	.906	<.001†	.002†

GQS, Global Quality Score; JAMA, *Journal of the American Medical Association* benchmark criteria; SD, standard deviation.

∗ Anatomy, clinical examination, surgical technique, and injury mechanism were grouped in one category. Bonferroni adjustment was used for pairwise comparisons.

† Statistically significant value.

Educational videos showed superior values (*P* < .05) for all scores compared with patient experience/testimony and greater values (*P* < .05) for DISCERN and GQS compared with rehabilitation videos. Detailed results are reported in Table 4. Videos with voice showed greater scores than video with a music background (*P* < .05).

**Table 4:** Score Difference by Video Content

				Pairwise Comparisons		
	Patient Experience/Testimony, n = 39	Rehabilitation, n = 31	Education, n = 30	Patient Experience/Testimony Versus Rehabilitation	Patient Experience/Testimony Versus Education	Rehabilitation Versus Education
	Mean ± SD	Mean ± SD	Mean ± SD	*P* Value	*P* Value	*P* Value
DISCERN	15.15 ± 0.54	18.23 ± 5.41	21.23 ± 5.95	<.001∗	<.001∗	.064
JAMA	0.03 ± 0.16	0.19 ± 0.48	0.57 ± 0.57	.137	<.001∗	.011∗
GQS	1.00 ± 0.00	1.10 ± 0.40	1.40 ± 0.56	.345	<.001∗	.018∗

NOTE. Bonferroni adjustment was used for pairwise comparisons.

GQS, Global Quality Score; JAMA, *Journal of the American Medical Association* benchmark criteria; SD, standard deviation.

∗ Statistically significant value.

## Discussion

The main finding of this study was that TikTok videos about the meniscus had poor reliability and validity and were found to be of low informational value. This was determined based on the DISCERN, JAMA, and GQS scores for the 100 reels analyzed. The DISCERN score, JAMA score, and GQS were extremely low, and none of them reached an average value of sufficient quality (DISCERN 17.93 ± 5.07, JAMA 0.24 ± 0.47, and GQS 1.15 ± 0.41).

Orthopaedic health care personnel should be conscious of the widespread availability of meniscus videos on TikTok and should highlight the limitations of the platform in disseminating accurate medical information. The quality of health-related content shared on social media is concerning because of the absence of standards governing the gathering, distribution, and promotion of information. Censorship in social media is a contentious topic, as these platforms frequently emphasize and benefit from their dedication to freedom of speech. Censorship and deplatforming are unlikely to reduce the spread of misinformation and may instead make it worse.^27^

The authors propose that a government entity, in collaboration with medical experts, create easily available, clear, and short informational material that is free for the general public. Although we cannot stop the creation of deceptive content, we are capable and responsible for offering this service to the public.

Recently, Kolade et al.^28^ evaluated the accuracy and popularity of content on common orthopaedic pathology, including meniscus, on TikTok and Instagram, concluding that common orthopaedic conditions such as Achilles tendon tears, ACL tears, and meniscus tears are frequently the focus of content posted on social media; however, this information is often not medically accurate. Similarly, D’Ambrosi and Hewett^29^ assessed the validity and informational value of TikTok videos with regard to the ACL and confirmed that the educational value of these videos is minimal.

Bethell et al.^30^ analyzed the quality, reliability, and educational value of TikTok videos among the patient population for ACL injury, hypothesizing that TikTok videos related to ACL rehabilitation exercises would lack quality, reliability, and educational value. The videos collectively received 335,577 likes, 2,969 comments, 22,856 favorites, and 6,142 shares. The tabulated scores for the DISCERN and ACL exercise education score (ACLEES) between general users and health care professionals were all statistically nonsignificant. Health care professionals had a greater percentage of videos with a “very poor” DISCERN score in comparison with the general public (66.67% vs 53.57%, respectively).

Hong et al.^31^ delved into the content present on TikTok that addresses the topic of knee OA, scrutinizing the makeup of content creators focusing on this subject and ascertaining whether a correlation exists between the viewership of a video and the strength of the recommendations it provides. The authors concluded that TikTok can be unreliable for knee OA treatment information. It is common to find nonphysicians sharing medical advice on the platform, with medical treatments demonstrating the weakest level of supporting evidence. Orthopaedic surgeons should advise their patients that TikTok treatment recommendations may not align with established guidelines.^31^

Finally, Jang et al.^32^ assessed the informational reliability, quality, and educational suitability of videos introducing scoliosis exercises on TikTok, revealing that the overall information quality, reliability, and educational suitability of videos on scoliosis exercises in TikTok appear to be low, suggesting that TikTok is not a suitable source for obtaining scoliosis exercise information.

Analyzing patient perception on TikTok, Aflatooni et al.^33^ evaluated posts on the social media platform, TikTok, to better understand the scoliotic patient condition. The authors found that more female posters post about scoliosis than male posters, with most posts containing positive self-image–related themes. This may represent a positive public attitude about scoliosis.^33^

Hand in hand with the development of social media and the internet, the concept of saving the meniscus has regained popularity in recent years, and as the internet still reports little or no educational information; likewise, there are still many artificial and incorrect reasons for orthopaedic surgeons to perform meniscectomies rather than meniscal repairs. One of the earliest beliefs concerns failure rate; when looking at the recent literature using modern devices, techniques, and selected indications, this rate is instead close to 7% to 10%. Another concern regards return to sport after meniscectomy versus meniscal repair; the recovery time for returning to sports is shorter after a meniscectomy. The effect of meniscectomy is more pronounced in the lateral meniscus because of the convex shape of the lateral tibial plateau. The shear stress is increased by 200% greater when the lateral meniscus is removed compared with medial meniscectomy.^34^, ^35^, ^36^

In the current era of social media and digital communication, it is intriguing to examine the decision-making process of patients when selecting a surgeon for disorders related to the ACL. Chapon et al.^37^ aimed to investigate the factors that influenced patients’ selection of surgeons for ACL restoration, a commonly performed treatment. The significance of the “human factor” in the doctor-patient contact was emphasized by the fact that 66% of patients acquired information about their surgeon through personal connections such as friends, family, or their primary care physician. Furthermore, effective communication, encompassing both spoken and written forms, must be unambiguous. Hence, the establishment of trust played a crucial role in the agreement forged between the physician and the patient.^37^

This is partially in contrast with the existing body of literature, as a growing number of patients are now seeking potential diagnoses online before visiting orthopaedic clinics, or conducting online research after their initial consultation. Most physicians commonly encounter patients who have previously conducted research on their medical problem online before their session. The patient-physician interaction is greatly affected by this, and 38% of physicians hold the belief that when a patient comes with preinformation, it diminishes the effectiveness of the session.^38^^,^^39^

Similarly, Subhash et al.^40^ tried to identify the information patients see after queries of Google search for orthopaedic surgeon providers. Most of the results were attributed to third-party websites, which demonstrates that orthopaedic surgeons do not have notable control over their digital footprint. Increased patient visibility of physician-controlled websites and an objective rating system for patients remain potential areas of growth.

### Limitations

There are limitations to this study. The search algorithm results can be modified by elements such as geographic location or user attributes. The analysis excluded movies in languages other than English, which further limited the generalizability of the current findings. The present study additionally employed reliability, validity, and quality evaluation instruments, namely DISCERN, JAMA, and GQS, whose complete validation has not been achieved. Nevertheless, these tools are extensively employed in research endeavors aimed at assessing the efficacy of these metrics for online services. A further limitation is the lack of comparison with other pathologies or other subspecialties (e.g., dermatology or plastic surgery) in order to better understand the educational value of these videos.

## Conclusions

Meniscus-related videos on TikTok are widely viewed and shared, but the overall educational value to patients is poor.

## Disclosures

All authors (R.D. and T.E.H.) declare that they have no known competing financial interests or personal relationships that could have appeared to influence the work reported in this paper.

## Supplementary Data

ICMJE author disclosure forms

ICMJE author disclosure forms
