# Supplementary material for: Meniscus-Related Videos on TikTok Are Widely Viewed and Shared but the Educational Quality for Patients Is Poor
Source: Arthrosc Sports Med Rehabil. 2024 Mar 19;6(3):100927. doi: 10.1016/j.asmr.2024.100927 (PMC11240010; doi:10.1016/j.asmr.2024.100927)
Supplement: ICMJE author disclosure forms [file mmc1.docx]

**Declaration of interests**
 
☒ The authors declare that they have no known competing financial interests or personal relationships that could have appeared to influence the work reported in this paper.
 
☐ The authors declare the following financial interests/personal relationships which may be considered as potential competing interests:

 
 
 
